# The global contribution of vultures towards ecosystem services and sustainability: An experts’ perspective

**DOI:** 10.1016/j.isci.2024.109925

**Published:** 2024-05-07

**Authors:** Andrea Santangeli, Sergio A. Lambertucci, Antoni Margalida, Tomaso Carucci, Andre Botha, Katherine Whitehouse-Tedd, Tommaso Cancellario

**Affiliations:** 1 Animal Demography and Ecology Unit, Institute for Mediterranean Studies (IMEDEA), CSIC-UIB, 07190 Esporles, Spain; 2 FitzPatrick Institute of African Ornithology, DST-NRF Centre of Excellence, University of Cape Town, Cape Town, South Africa; 3 Grupo de Investigaciones en Biología de la Conservación, INIBIOMA, Universidad Nacional del Comahue - CONICET, Quintral 1250 (R8400FRF), Bariloche, Argentina; 4 Institute for Game and Wildlife Research, IREC (CSIC-UCLM), Ronda de Toledo, 12. 03005, Ciudad Real, Spain; 5 Pyrenean Institute of Ecology (CSIC), Avda. Nuestra Señora de la Victoria 16, 22700 Jaca, Spain; 6 School of Animal, Rural and Environmental Sciences, Nottingham Trent University, Southwell, UK; 7 Endangered Wildlife Trust, Midrand, Gauteng 1685, South Africa; 8 Balearic Biodiversity Centre, Department of Biology, University of the Balearic Islands, 07122 Palma, Spain

**Keywords:** Nature conservation, Ecology, Environmental resource, Ornithology

## Abstract

The ecosystem services framework is essential for biodiversity conservation, emphasizing the role of nature in achieving sustainable development goals (SDGs). This study offers a global view on vulture-associated ecosystem services and their SDG contributions, based on insights from 206 experts. The findings reveal global consensus on the importance of vultures in regulation and maintenance services, such as waste recycling and disease control. Cultural services attributed to vultures are moderate and vary regionally. Provisioning services are consistently rated low across all regions. Experts’ views on vultures' key ecosystem roles are often biased toward well-known services and may not align with all scientific evidence. The study emphasizes vultures’ role in achieving SDGs, particularly impacting life on land and health, and calls for reevaluating their contribution to sustainable practices. It stresses the need to customize conservation to regional values and perceptions, recognizing vultures’ critical role in ecological balance, public health, and sustainable development.

## Highlights

• Global expert consensus on vulture’s regulation and maintenance services
• Vulture’s cultural services are moderate and provisioning ones are low
• High vulture’s role toward SDGs life on land & health and sanitation
• Vulture’s play a key role in biodiversity conservation and sustainability

## Graphical abstract

**Figure:**
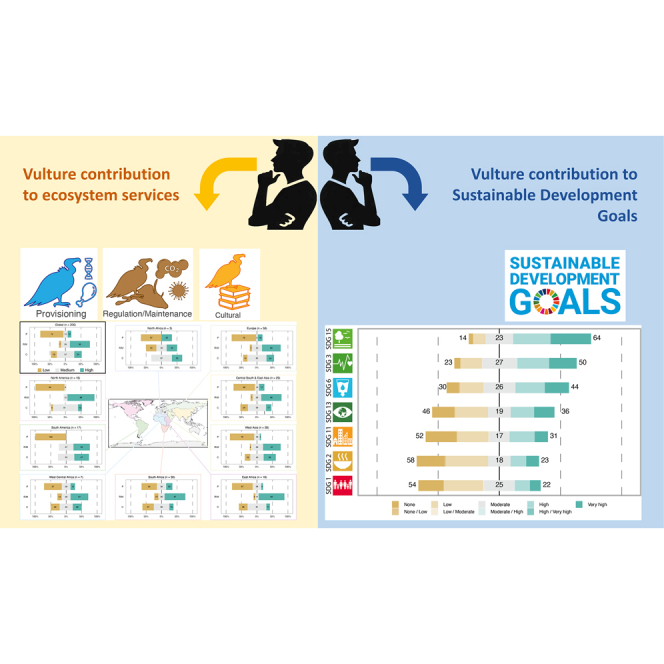

## Introduction

Biodiversity and ecosystem services are the main pillars of the life-support system, ultimately allowing human societies to thrive.^1^ The unprecedented global decline of biodiversity, with an estimated one million species being threatened with extinction, calls for a rapid transformative change.^2^ Promoting this change can be achieved not only by international conservation policies and targets, such as the sustainable development goals (SDGs) but also by recognizing and leveraging the importance and value of ecosystem services associated with biodiversity and its conservation.^1^

The concept of ecosystem services has often been associated with distinct groups of species, such as pollinators, to catalyze conservation policy attention toward such groups.^3^ Birds are also been often associated with various ecosystem services.^4^^,^^5^^,^^6^ Specifically, the generally positive relationship between bird abundance and ecosystem service provision highlights the relevance of conserving bird populations in order to preserve the associated ecosystem services they provide to human societies.^6^ For example, correlative and post-hoc evidence suggests that the collapse of vultures in parts of Asia coincided with an increase in feral dog populations and resulting rabies burden to the society.^7^^,^^8^ In this case, the hypothesis, yet to be conclusively and experimentally tested,^9^ is that the cleaning service (waste disposal) provided by the avian scavengers went missing, and large amounts of organic waste then became available to feral dogs and other facultative scavengers. These opportunistic scavengers then grew in numbers, boosting the spread of diseases such as rabies.^7^^,^^8^ Over the years, the regulation and maintenance service provided by vultures in particular has somewhat become established within the community of vulture experts, scientists and conservationists, and among the public, despite existing scientific evidence being rather limited.^10^^,^^11^^,^^12^ Similarly, cultural connections relating vultures to spiritual and religious ceremonies do exist, but this information is mostly preserved in local peoples’ traditional knowledge.^13^^,^^14^^,^^15^

In the field of ecology and nature conservation, experts may play an important role in generating, filtering, and disseminating scientific information, especially in an era dominated by social media and the rapid spread of misinformation. Their role extends beyond research, as they critically evaluate and interpret complex ecological data, ensuring its integrity and relevance in public and policy discourse. Public opinion on nature is often shaped by the advocacy and messages derived from the experts through their knowledge and perceptions.^16^ However, expert opinions are not always free from cognitive and other bias sources that may hamper accuracy and reliability.^17^ Nevertheless, expert knowledge can be used to understand how conservation policies align with other targets, such as sustainability,^18^ as well as to identify conservation priority actions.^19^ In each case, it is fundamental to also quantify how expert opinions align with scientific evidence and critically scrutinize the role of experts in conservation. In the case of vultures, expert knowledge has been recently harnessed in order to develop a multi-species action plan to conserve African-Eurasian vultures,^20^ and to aid in the identification of priority areas for Old World vulture conservation.^21^

The popularity of the idea that vultures are “nature’s clean-up crew” has grown enormously over the past decade (e.g.,^22^^,^^23^ with less attention being given to other contributions, such as cultural services (e.g., spiritual). Moreover, preserving ecosystem services is an integral part of achieving sustainable development.^24^ In the case of vultures, the ecosystem services they provide may align with the achievement of specific sustainable development goals. Moreover, given the very large areas that vultures can cover, such services span far beyond administrative borders.^23^^,^^25^ Globally there are 23 species of vultures and condors widely distributed across the New World, from South to North America,^26^ and the Old World,^21^ from across most of Africa, to the southern half of Eurasia (excluding Australia).

A systematic review of the scientific evidence underpinning the ecosystem services provided by vultures was recently published^12^ and highlighted large gaps in the scientific literature on this topic. Expert knowledge often reveals critical ecological insights, as evidenced in the role of vultures in disease control. Therefore, expert opinion could be valuable in complementing existing literature and the gaps thereof. To date, we still lack a global understanding of how experts perceive the ecosystem services provided by vultures, as well as how vultures may contribute to achieving the SDGs. Therefore, here we quantify the contribution of vultures toward different ecosystem services and toward key sustainable development goals as perceived by experts. Specifically, using expert scores we: (1) quantify the perceived extent to which vultures can provide key ecosystem services, and whether this varies regionally; (2) quantify the perceived strength of scientific evidence supporting the ecosystem service provision by vultures, and we compare this with the evidence available through a recent systematic review of the scientific literature; (3) investigate the factors affecting experts’ scoring of the scientific evidence supporting the ecosystem service provision by vultures; and (4) quantify the expert-derived relevance of vultures toward addressing key sustainability issues (i.e., SDGs).

## Methods

We obtained the list of ecosystem services from the Common International Classification of Ecosystem Services (CICES V5.1,^27^; which represents the most recent and authoritative international classification of ecosystem services (hereafter ES). The CICES classification focuses on describing the contribution that ecosystems make to human well-being, essentially defining “what ecosystems do”. Being centered on the ecological outcomes generated by an ecosystem and that can ultimately benefit people, CICES aims to classify the multiple purposes or uses that people have of various ecosystem services. CICES follows a hierarchical classification structure, with the broadest and more general categories followed by more specific nested levels.^27^ For this study, we considered two of the CICES classification levels for ES. The broadest level, named “section” in the CICES database, includes three categories: provisioning, regulation and maintenance, and cultural.^27^ We also considered a more specific level named “class” in CICES. Class defines 90 specific and unique categories of ecosystem services in CICES (see Table S1). We thus screened through all the 90 ecosystem services listed as CICES classes to identify *a priori* those that could be of any relevance to vultures, following the approach of Carucci et al.^12^ For this step, we took a conservative approach to minimize the risk of excluding classes that could be of relevance. We first excluded all classes related to non-animals and non-terrestrial realms and all those related to abiotic services (i.e., services provided by abiotic means, like water). Next, we screened through the remaining classes and identified a manageable number of 19 classes, three for provisioning, seven for regulation and maintenance, and seven for cultural services (Table S1). Similarly, we identified among the 17 sustainable development goals those that could *a priori* be, at least in some way, associated with vultures. This screening resulted in the identification of 7 SDGs (Table S2).

In order to address the aims of this study, we devised an online questionnaire (see appendix S1) that was distributed to vulture experts (including scientists, practitioners, and other groups with at least one year of experience working with vultures) worldwide via personal contacts, snowball effects on social media, as well as through mailing lists of the Convention on Migratory Species Raptors MoU, the IUCN SSC Vulture Specialist Group and the Vulture Conservation Foundation. The questionnaire included some socio-demographic questions, such as age, organization, level of education, occupation, region of work, number of years that respondent has been working with vultures and primary expertise. Next, it asked respondents to rank the relevance to vultures of each of the 19 classes of ecosystem services (relevant to aim 1) and the seven SDGs (relevant to aim 4) using a 5-point Likert scale, from none, low, moderate, high, or very high relevance. Here, respondents could also choose multiple options, e.g., “high” and “very high”. It then asked to rank the relevance of each of the three ecosystem services main group (provisioning, regulation/maintenance, and cultural) according to a scale with three levels, from least, medium, and most relevant (relevant to aim 1). Finally, it asked respondents to score (using a 9-point Likert scale: from “No evidence” to “very strong”) the strength of the scientific evidence underpinning the association of vultures to each of the three ecosystem services groups (relevant to aim 2 and 3). The questionnaire was made available online (using Google forms) in spring 2020 and was left open for three months. Each respondent was initially given information about the study, and informed consent was obtained from every respondent, who could drop out without submitting at any stage of the questionnaire filling. We did not collect any personal identification information, but respondents were asked to voluntarily write their email address at the end of the survey if they were interested in receiving updates on this study. We processed the data according to the privacy policy of the Finnish Biodiversity Information Facility.^28^ Prior to administering the questionnaire to potential respondents, we pilot tested it with a sample of three colleagues to test the clarity and comprehensibility of the questions. The content validity of the questionnaire was ensured by selecting lists of questions directly from existing classifications of ecosystem services and sustainable development goals. This enables direct associations of the scores to each question with the associated ecosystem service or sustainable development goal.

Respondents’ scores on the perceived strength of evidence supporting the ecosystem service provision by vultures were then compared to the recently quantified scientific evidence (aim 2; see in the following text). The latter was assessed in a recently published systematic review of the literature of all scientific publications on ecosystem services and disservices associated with vultures.^12^ The aforementioned review was based on an initial literature search followed by an evidence scoring of the scientific basis underpinning the association between vultures and each of the three ecosystem service groups (provisioning, regulation/maintenance, and cultural) by employing a modification of the framework proposed by.^29^

In order to test the effect of socio-demographic features of respondents on their assessment of the scientific evidence underpinning the ecosystem services provided by vultures, we used a linear mixed model (using the lme function from the lme4 package in R;^30^). This model included as the response the scientific evidence score. This score was originally given on a 9-point Likert scale (from “No evidence” to “Very strong evidence”) and converted to a numerical score from 0 (“No evidence”) to 8 (“Very strong evidence”). Each respondent separately scored each of the three ecosystem service groups, so that one sample unit of the response was the score for each ecosystem service group by each respondent. We thus included the respondent identity as a random factor to account for pseudoreplication resulting from three assessments of scientific evidence, one per each ecosystem service group, by each respondent. As predictors, we included ecosystem service group type (three classes: provisioning, regulation/maintenance, and cultural), region of work (nine classes), and number of years the respondent has been involved in vulture work (continuous variable). We also included the average score given for the relevance of each of the three ecosystem service groups to vultures (converted to a numeric variable from the 5-point Likert scale) and six categorical variables each depicting a listed main field of expertise by the respondent: whether the respondent listed conservation, ecology or biology, agriculture, education, forensic or veterinary, or social sciences (yes or no). While region of work was mainly included to control for spatial patterns not of direct interest here, we tested the number of years a respondent worked on vultures under the rationale that longer involvement may result in increased knowledge, or perception, of the scientific evidence linking vultures to ecosystem services. The model results were validated by conducting a residual exploration analysis. All assumptions, including collinearity and residual normality, were met.

The relevance of different ecosystem services and SDGs to vultures and the scientific evidence underpinning the link of vultures to ecosystem services and SDGs are presented as descriptive plots created using the Likert package in R.^31^ R software version 4.0.3 was used for all the modeling and for figure preparation.

## Results

### Respondent’s information

**Figure 1:**
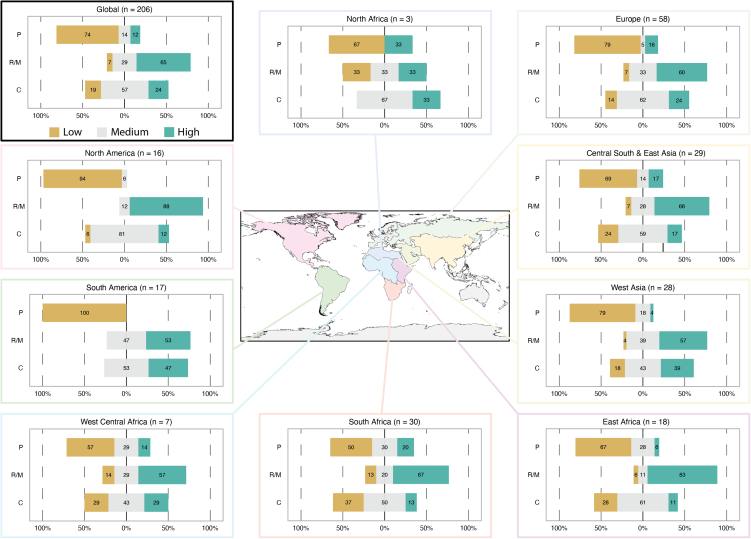
The perceived relevance of each of three main ecosystem service groups (P - provisioning, RM - regulation and maintenance, C - cultural) to vultures as assessed by experts at the global (top-left panel) and regional (other panels) level mk The number of respondents per each plot is given at the top along with the region name. The regions for Africa–Eurasia follow those used in (Botha et al. 2017). Numbers within each panel represent the % frequency of each scoring scale for a particular ecosystem service group (low, medium or high, from left to right).

A total of 206 vulture experts filled the online questionnaire, with a good global representation (Figure 1). Average age of respondents was 44 years, 29% females, with an average of 10 years of experience working with vultures. With regards of the primary field of expertise, 81% of respondents associated it to conservation, 66% to ecology/biology, 17% to education, 11% to veterinary/forensic, 5% to social sciences, and 3% to agriculture (these fields are not mutually exclusive, e.g., one respondent could list one or multiple fields of expertise).

### Vulture’s association to ecosystem service

At a global scale, regulation and maintenance services were considered at the highest relevance to vultures (65% of scores), followed by cultural which had medium relevance (57% of the scores), whereas the majority of experts (74%) assigned a low relevance to provisioning services (Figure 1). Variation in the expert-assessed relevance of each ES group to vultures was high across the various regions considered. While regulation and maintenance had high relevance across all regions, cultural services varied widely, being highest for some regions, like West Asia and South America, and medium in most other regions (Figure 1). The relevance of provisioning services was consistently low across most regions, particularly in the Americas.

**Figure 2:**
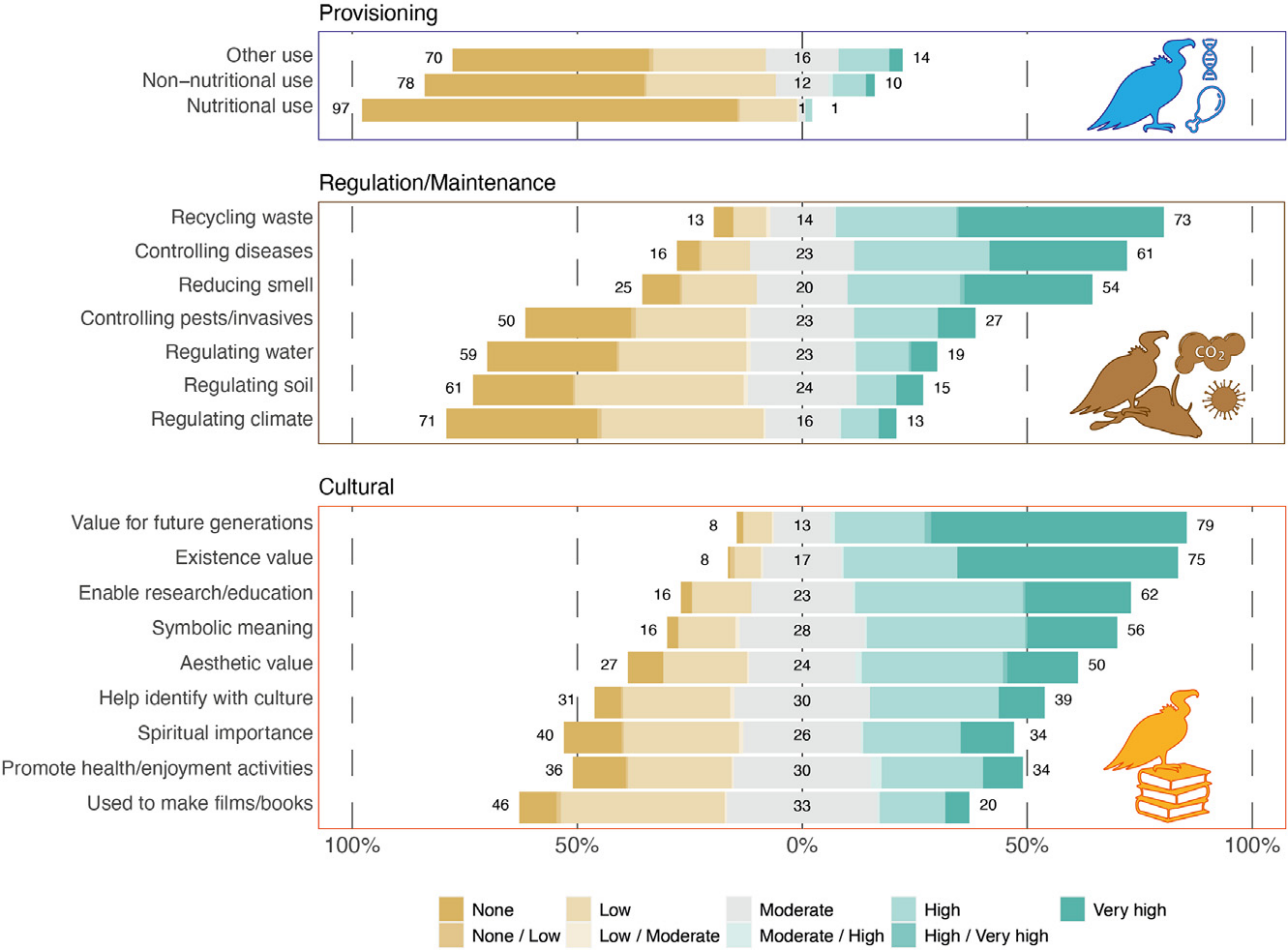
Ecosystem services associated to vultures The overall potential relevance of ecosystem services provided by vultures for each specific ecosystem service class within the main groups (regulation and maintenance, cultural, and provisioning) as assessed by 206 experts globally.

We also show a large variation among specific ecosystem service classes within each of the three groups considered (Figure 2). Among cultural services, the value for future generation, existence value, services related to enabling research and education as well as symbolic meaning were scored high or very high by over 50% of respondents (Figure 2). Large variation was also apparent among regulation and maintenance services, with recycling waste, controlling disease and reducing smell scored high or very high by over 50% of respondents (Figure 2). Conversely, all the three provisioning service classes were scored with low relevance by most respondents.

### Alignment of expert assessments of vulture ecosystem services with existing evidence

**Figure 3:**
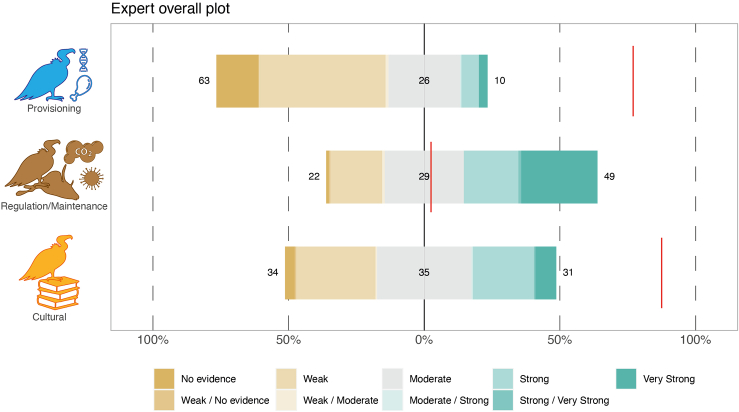
The overall strength of the evidence underpinning the relevance of ecosystem services provided by vultures for each ecosystem service group (regulation and maintenance, cultural, and provisioning) as assessed by 206 experts globally Values to build this figure are derived from the raw data. The red lines for each ecosystem service group depict the level of scientific evidence score as derived from a systematic literature review and evidence scoring (Carucci et al. 2022).

The strength of the scientific evidence underpinning the potential relevance of ecosystem services provided by vultures, as scored by experts, was related to several factors (Table 1). Specifically, evidence varied by ecosystem service group, being significantly higher (*p* < 0.001 after post-hoc testing with Tukey adjustment, see Table S3) for regulation and maintenance compared to cultural and provisioning services (Table 1; Figure 3). We found a positive correlation between the number of years that experts worked with vultures and the scores they assigned to the evidence supporting ecosystem service associated to these birds. Moreover, experts who provided a higher score for the relevance of the ecosystem service associated to vultures also gave a higher score for the underlying scientific evidence (Table 1). None of the fields of expertise of the respondent had any strong significant effect on the evidence scoring.

**Table 1:** The factors related to variation in expert scoring (the response variable in the model, values ranging from 0 lowest to 8 highest) of the scientific evidence underpinning the relevance of ecosystem services provided by vultures

	Coeff	SE	t	F	*p*-value
Intercept	4.35	0.38	11.41		<0.001
ES group (3 classes)				91.37	<0.001
Cultural	−1.45	0.18	−8.04		<0.001
Provisioning	−1.21	0.22	−5.47		<0.001
Region (9 classes)				4.57	<0.001
N. years working with vultures	0.03	0.01	2.64		0.009
Expertise - Conservation	0.18	0.24	0.73		0.469
Expertise - Ecology/Biology	0.06	0.20	0.31		0.759
Expertise - Agriculture	−1.14	0.58	−1.98		0.049
Expertise - Education	−0.20	0.25	−0.78		0.435
Expertise - Veterinary/Forensic	0.59	0.32	1.83		0.069
Expertise - Social sciences	0.18	0.42	0.42		0.675
ES relevance score to vultures	0.45	0.05	8.61		<0.001

Coefficient and SE, along with t statistics and *p*-value are shown for all variables. For ES group, regulation and maintenance class serves as the reference category, whereas for the six expertise variables, the “no” category serves as a reference. For simplicity, and because the region was only controlled for in the model, we here omit the results of each region class. ES relevance score to vultures depicts the average numeric score the respondent gave to the relevance of vultures toward the ES group (as shown in Figure 1). Overall F statistic and *p*-value are reported also for the two categorical variables (ES group and Region) with more than two classes. Post-hoc test results for all combinations of classes within the ES group and region are shown in Table S3).

### Vulture’s contribution to sustainable development

**Figure 4:**
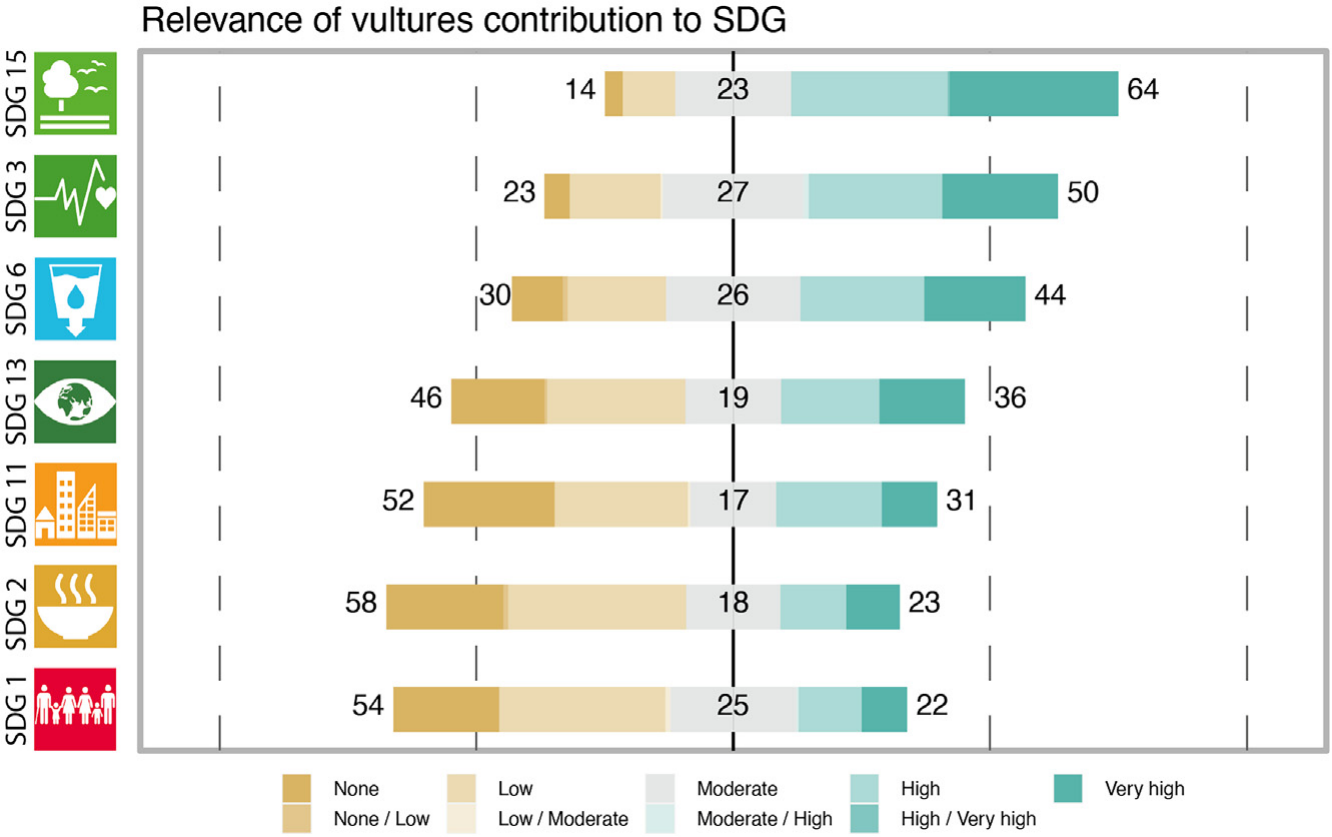
Vultures contribution to sustainability goals The relevance of vultures (upper plot) toward achieving each of the 7 selected sustainable development goals (SDG, the number next to each SDG in the y axis represents the original SDG number as listed by the United Nations) as scored by 206 vulture experts globally. SDG 15 - Life on land, SDG 3 - Good health/well-being, SDG 6 - Clean water/sanitation, SDG 13 - Climate action, SDG 11 - Sustainable cities/communities, SDG 2 - Zero hunger, SDG 1 - No poverty.

The perceived contribution of vultures toward sustainable development goals was strongest for SDG15 life on land and SDG3 good health and sanitation, with 64 and 50% of respondents scoring them as moderate/high to very highly relevant, respectively (Figure 4). SDG6 clean water and sanitation was also perceived as relevant (44% scores from moderate/high to very high compared to 30% scores from moderate/low to none). The other four SDGs were deemed as largely moderately to low or not relevant by the majority of respondents.

## Discussion

Our study, involving 206 vulture experts globally, reveals important insights into the perceptions of ecosystem services provided by vultures and their alignment with sustainable development goals (SDGs). The findings indicate that vultures are predominantly associated with regulation and maintenance services, as well as cultural services, with the latter varying regionally, being of highest relevance in West Asia and South America. In contrast, provisioning services are consistently deemed of low relevance across most regions. The experts’ assessment of the evidence supporting vulture ecosystem service associations was positively related to the number of years they worked with vultures and also to their scored relevance of vulture ecosystem services. According to the experts, vultures have potential to contribute substantially to SDG15 (Life on Land), SDG3 (Good Health and Well-being), and SDG6 (Clean water/sanitation), while their contribution to other SDGs is perceived as less important.

### Vultures’ ecosystem service contribution

The global consensus on the high contribution of vultures toward regulation and maintenance services largely aligns with the widespread and popularized view of vultures as natures’ clean-up crew, owing to their scavenging role in the ecosystem.^22^^,^^23^^,^^32^ This is also confirmed by the high score given to specific ecosystem service classes, such as recycling waste, controlling disease, and reducing smell. These contributions are highlighted not only in scientific studies but also among the general media, reaching millions of people globally, and became crystallized in the general public, conservation practitioners, and also scientists’ perception.^7^^,^^9^ This may have triggered a cognitive bias in the persons exposed to such information, including experts. As a result, it is not surprising that two-thirds of experts scored regulation and maintenance services as high.

Cultural ecosystem services were assigned medium relevance in relation to vultures by over half of the experts globally, but this pattern varies regionally. For example, vultures’ contribution as cultural services was deemed highest in South America, followed by West Asia, North Africa, West-Central Africa, and Europe. In South America, for example, the majestic Andean Condor (*Vultur gryphus*) is the national bird of Chile, Ecuador, Colombia, and Bolivia, it contributes ecotourism revenue in various sites, such as the Cruz del Condor in the Peruvian Andes,^33^ and represents a biocultural keystone species for traditional Andean societies.^34^ Similarly, Griffon vultures (*Gyps fulvus*) were found to contribute large ecotourism revenues in Israel’s (West Asia) nature reserves,^35^ and in two regions of the Spanish Pyrenees, where annual ecotourism revenues exceeded two million € annually.^36^ In parts of West Africa, the Hooded vulture (*Necrosyrtes monachus*) holds spiritual values,^37^ as do other vulture species in Asia through sky burials and other traditional meanings.^13^ However, we note that this assessment of cultural ecosystem services may be biased due to the experts being working with vultures, thus being potentially and subconsciously biased.

Interestingly, for the Central South and East Asia region, more experts scored the cultural services associated with vultures as low. This finding seems in apparent contrast with the popularized image of vultures in the traditional spiritual culture of the people in these regions.^13^ During the last decades, however, the regulation and maintenance services (waste disposal and/or disease control) contributed by vultures in Central South and East Asia have become very prominent, following a seminal study from India^7^ that was widely popularized among the general public and scientific communities. The dominance of this service contribution by vultures in India, and the rest of Central East Asia, may have obscured that of other likely important services, such as the cultural ones, thereby explaining the findings of this study. Among the specific ecosystem services classes within the cultural services, the value for future generations and existence values were given the highest relevance by experts. This underscores the important intrinsic non-material contribution of vultures which goes beyond any economic and utilitarian valuation and closely aligns with the modern concept of nature’s contribution to people.^38^

The provisioning services contributed by vultures are scored as low globally, with this pattern largely consistent across the regions. While minimal, provisioning services are, in most cases, related to the use of body parts of vultures, such as feathers or skull, for traditional medicinal practices largely occurring in Africa and Asia.^12^

The strongest perceived scientific evidence associated with the regulation and maintenance contribution by vultures found here seems to contrast with available scientific evidence. Indeed, a recent literature review found only weak scientific evidence support for the regulation and maintenance services contributed by vultures.^12^ This mismatch is likely due to the widespread popularity of the vultures waste disposal and disease regulation services, which became pervasive also among experts, ultimately shaping their valuation. This conclusion is also supported by the model results showing that experts who assign a higher relevance score to a specific ecosystem service group, also tend to give to that service a higher estimate of the evidence strength. This result may also suggest that not all experts critically scrutinize the evidence strength (e.g., correlative versus experimental) of scientific findings. Moreover, experts seem to give a higher evidence strength score to each ES group associated with vultures when they worked longer with vultures. This is likely explained by their growing knowledge of the scientific literature on the topic or their anecdotal experience with the system. Overall, the mismatch between the expert assessment and the evidence stemming from scientific literature suggests caution in using expert opinion at the interface of biodiversity and ecosystem services. Indeed, it is known that expert opinion may be compromised by cognitive biases,^17^ such as confirmation bias, whereby they may tend to favor information that confirms previous beliefs or biases. The aforementioned mismatch thus calls for the need to gather the scientific evidence to be able to achieve effective conservation policy.^39^

### Vultures’ contribution to sustainable development goals

The association of vultures to key sustainable development goals (SDGs) in our expert-based study reveals their critical ecological and societal role. Specifically, their strong alignment with SDG15 (Life on Land) and SDG3 (Good Health and Well-being) highlights their importance in biodiversity conservation and ecosystem balance across the different spheres of the life-support system. As obligate scavengers, vultures play an indispensable role in carrion decomposition, potentially contributing to maintaining ecological health and preventing disease spread.^7^^,^^8^^,^^40^^,^^41^^,^^42^ This is even more salient given the reduced number of vulture species (only 23) present worldwide. Furthermore, their indirect role in supporting SDG6 (Clean Water and Sanitation) by preventing water contamination through carrion decomposition is an area that warrants greater exploration and acknowledgment. Taken together, the aforementioned associations underscore the multifaceted ecological services vultures provide, which are crucial for achieving several key SDGs, particularly in the context of environmental sustainability and public health.

In the Anthropocene, an era defined by significant human influence on the planet, the role of vultures transcends ecological boundaries, impacting human societies and overall sustainability. Their ecological services are not only pivotal for maintaining biodiversity and ecosystem health but also have far-reaching implications for human well-being and sustainable development. The decline in vulture populations, therefore, may pose a substantial risk to achieving sustainable development goals, highlighting the need for integrated conservation strategies. By conserving vultures, we not only protect an essential component of the ecosystem but also preserve a critical pillar of our life-support system. This understanding emphasizes the need for a holistic approach in conservation efforts, where the protection of species like vultures is intricately linked to the broader goal of sustaining human societies and ensuring a healthy, resilient planet for future generations.

It is important to acknowledge that this study may carry some limitations. The questionnaire was only delivered in English language, thus captured only experts that are more internationalized, which at the same time may also have more access to the scientific literature in English. Moreover, we did not account for the place of residence of the experts but only for the place where they work on vultures. As a result, the results may not necessarily capture local expert overviews, but mainly the overview of experts that, at least for one or likely more years, have worked with vultures in the region. Moreover, we considered all vulture species as a single guild, the obligate avian scavengers, as we were interested in a global perspective relating to this guild. Further studies may investigate species-specific patterns of vulture associations to ecosystem services and sustainable development goals. Finally, we did not collect information on the religion followed by experts, which is known to relate to some critical ecosystem services, such as cultural ones. We believe that the region of work, which we used here, may partly capture religion as well.

### Concluding remarks and recommendations

Overall, through the eyes of experts worldwide, we highlight vultures’ key contribution to people, with potential positive impacts also toward achieving several sustainable development goals, underscoring their value in sustainable development efforts worldwide. These findings advocate for the adoption of a One Health approach in vulture conservation, an approach that recognizes the interconnectedness of human, animal, and environmental health.^43^^,^^44^ By integrating conservation strategies for vultures with public health and environmental policies, we can create synergistic benefits that extend beyond ecological boundaries, enhancing societal well-being and global sustainability. This holistic approach is particularly crucial in the Anthropocene, where human-induced environmental changes demand innovative and integrated solutions. The conservation of vultures, therefore, is not just an ecological imperative but a necessary strategy for fostering resilient and sustainable human societies, highlighting the profound and often underappreciated role these birds play in the health of our planet.

## STAR★Methods

### Key resources table

**Table:** 

REAGENT or RESOURCE	SOURCE	IDENTIFIER
**Deposited data**		

Raw and Analyzed Data	This Paper	N/A

**Other**		

Online Questionnaire	This Paper	N/A

### Resource availability

#### Lead contact

Further information and requests for resources and reagents should be directed to and will be fulfilled by the lead contact, Andrea Santangeli (andrea.santangeli@gmail.com).

#### Materials availability

The Questionnaire is available in the Supplementary Materials of this paper.

#### Data and code availability

• All data reported in this paper will be shared by the lead contact upon request.
• This paper does not report original code.
• Any additional information required to reanalyze the data reported in this paper is available from the lead contact upon request.

### Experimental model and subject details

Ethical approval was not needed, as outlined in the manuscript. All participants in the study provided their informed consent prior to completion of the questionnaire.

### Method details

We utilized the Common International Classification of Ecosystem Services (CICES V5.1) to categorize ecosystem contributions to human well-being.^45^ For this study, we focused on two hierarchical levels of CICES: broad "sections" (provisioning, regulation and maintenance, cultural) and more specific "classes" (90 unique categories). We screened these classes to select those relevant to vultures, excluding non-animal and abiotic classes, resulting in 19 relevant classes. Concurrently, we identified 7 Sustainable Development Goals (SDGs) potentially linked to vultures.

An online questionnaire was developed to assess the relevance of these ecosystem classes and SDGs among vulture experts worldwide, using distribution via professional networks and social media. The survey, hosted on Google Forms in spring 2020 for three months, asked respondents to rank the relevance of ecosystem service classes and SDGs on a 5-point Likert scale and to assess the scientific evidence supporting these associations on a 9-point scale. Privacy was ensured according to the Finnish Biodiversity Information Facility’s policies,^28^ and content validity was checked through pilot testing with colleagues.

The perceived strength of evidence from respondents was then compared to actual scientific evidence from a systematic literature review on vulture-associated ecosystem services and disservices.^12^

### Quantification and statistical analysis

To explore the influence of socio-demographic characteristics on respondents' ratings of scientific evidence for vulture-related ecosystem services, we applied a linear mixed model using the lme function from the lme4 package in R.^30^ The model assessed the numerical conversion (0–8) of a 9-point Likert scale score given to each of three ecosystem service groups (provisioning, regulation/maintenance, cultural). Respondent identity was incorporated as a random factor to address pseudoreplication from multiple assessments by the same individuals.

Predictor variables included the type of ecosystem service group, respondent’s region of work, years of vulture-related experience, relevance scores for ecosystem service groups (converted from a 5-point Likert scale), and categorical variables representing fields of expertise (conservation, ecology, biology, agriculture, education, forensic/veterinary, social sciences). The model controlled for spatial patterns via region of work and tested the impact of duration in vulture-related work on knowledge or perception of scientific evidence.

Model validation involved residual exploration analysis to confirm assumptions like collinearity and normality of residuals were met. Results and the relationship between ecosystem services, SDGs, and vultures were visualized using the likert package in R, with all analyses performed in R version 4.0.3.

## Supplemental information

Document S1. Figures S1‒S3

Document S2. SM_Appendix_S1_Online_Questionnaire
