# Supplementary material for: The global contribution of vultures towards ecosystem services and sustainability: An experts’ perspective
Source: iScience. 2024 May 7;27(6):109925. doi: 10.1016/j.isci.2024.109925 (PMC11112611; doi:10.1016/j.isci.2024.109925)
Supplement: Document S1. Figures S1‒S3 [file mmc1.pdf]

iScience, Volume *27*

Supplemental information

The global contribution of vultures

towards ecosystem services

and sustainability: An experts' perspective

Andrea Santangeli, Sergio A. Lambertucci, Antoni Margalida, Tomaso Carucci, Andre Botha, Katherine Whitehouse-Tedd, and Tommaso Cancellario

Table S1 (Relates to Figure 1 and 2). List of the selected 19 ecosystem service classes with relevance to vultures as derived from CICES V5.1 ecosystem service classification and selected a priori from the full list of classes of ecosystem services. The ecosystem service group level (Section), as well as the class levels with relevance to vultures are reported, as well as the original CICES ecosystem service class code and simple descriptor. Note that two of the classes used for this study resulted from the pooling of multiple CICES classes due to their similarity in the services their represent and their interpretation.

**Section**	**Class - with relevance to vultures**	**CICES code**	**CICES Simple descriptor**
Provisioning	Vulture, or their body parts, used for nutritional purposes	1.1.6.1	Food from wild animals
Provisioning	Vulture, or their body parts, used for non-nutritional purposes	1.1.6.2	Materials from wild animals
Provisioning	Vultures used for replenishing stock, for breeding, or for genetic stock that can have a value for humans	1.2.2.1 & 1.2.2.2 & 1.2.2.3	Animals used for replenishing stock & Wild animals that we can use for breeding & The genetic information that is stored in wild animals that we can use
Regulation & Maintenance	Vultures aid in decomposing waste	2.1.1.1	Decomposing wastes
Regulation & Maintenance	Vultures aid in reducing smell	2.1.2.1	Reducing smells
Regulation & Maintenance	Vultures aid in controlling pests and invasive species	2.2.3.1	Controlling pests and invasive species
Regulation & Maintenance	Vultures aid in controlling disease	2.2.3.2	Controlling disease
Regulation & Maintenance	Vultures aid in regulating soil quality	2.2.4.2	Ensuring the organic matter in our soils is maintained
Regulation & Maintenance	Vultures aid in regulating water conditions	2.2.5.1	Controlling the chemical quality of freshwater
Regulation & Maintenance	Vultures aid in regulating the global climate	2.2.6.1	Regulating our global climate
Cultural	Vultures enable activities promoting health (e.g. reduce stress) and enjoyment	3.1.1.2	Watching plants and animals where they live; using nature to de-stress
Cultural	Vultures enable the advance of research, knowledge and education on the natural world	3.1.2.1 & 3.1.2.2	Researching nature & Studying nature
Cultural	Vultures help people identify with the history or culture of where they live or come from	3.1.2.3	The things in nature that help people identify with the history or culture of where they live or come from
Cultural	Vultures have aesthetic value	3.1.2.4	The beauty of nature
Cultural	Vultures have symbolic meaning	3.2.1.1	Using nature to as a national or local emblem
Cultural	Vultures have spiritual importance for people	3.2.1.2	The things in nature that have spiritual importance for people
Cultural	Vultures are used to make films or to write books	3.2.1.3	The things in nature used to make films or to write books

Cultural	Vultures have an existence value, we think they should be conserved	3.2.2.1	The things in nature that we think should be conserved
Cultural	Vultures should be conserved for the future generations to enjoy or use	3.2.2.2	The things in nature that we want future generations to enjoy or use

Table S2 (Relates to Figure 4). List of the selected 7 Sustainable Development Goals of the United Nations (www.sdgs.un.org/goals) with relevance to vultures and selected a priori from the full list of goals.

**Sustainable development goals:**	**Description:**
GOAL 1: No Poverty	Eradicating poverty in all its forms. This involves targeting the most vulnerable, increasing basic resources and services, and supporting communities affected by conflict and climate-related disasters.
GOAL 2: Zero Hunger	End all forms of hunger and malnutrition, making sure all people–especially children–have sufficient and nutritious food all year. This involves promoting sustainable agricultural, supporting small-scale farmers and equal access to land, technology and markets.
GOAL 3: Good Health and Well-being	Good health is essential to sustainable development and the 2030 Agenda reflects the complexity and interconnectedness of the two. It takes into account widening economic and social inequalities, rapid urbanization, threats to the climate and the environment, the continuing burden of HIV and other infectious diseases, and emerging challenges such as noncommunicable diseases. Universal health coverage will be integral to achieving SDG 3, ending poverty and reducing inequalities.
GOAL 6: Clean Water and Sanitation	Safe and affordable drinking water for all by 2030 requires we invest in adequate infrastructure, provide sanitation facilities, and encourage hygiene. Protecting and restoring water-related ecosystems is essential.
GOAL 11: Sustainable Cities and Communities	Making cities sustainable means creating career and business opportunities, safe and affordable housing, and building resilient societies and economies. It involves investment in public transport, creating green public spaces, and improving urban planning and management in participatory and inclusive ways.
GOAL 13: Climate Action	Address the needs of developing countries to both adapt to climate change and invest in low-carbon development.
GOAL 15: Life on Land	Human life depends on the earth as much as the ocean for our sustenance and livelihoods. Urgent action must be taken to reduce the loss of natural habitats and biodiversity which are part of our common heritage and support global food and water security, climate change mitigation and adaptation, and peace and security.

Table S3 (Relates to Figure 3 and Table 1). Results of the post-doc testing (applying the Tukey correction for multiple testing) of all combination among ecosystem service groups and among regions in affecting the strength of the evidence underpinning the relevance of ecosystem services provided by vultures. These results are derived from the model presented in Table 1 and Figure 3 in the main manuscript. Significant pair combinations are highlighted in grey and the p-value presented in bold font.

ES group pairs	Estimate	SE	df	t	p
(Regulation/Maintenance)-Cultural	1.446	0.180	371	8.044	**<.0001**
(Regulation/Maintenance)-Provisioning	1.208	0.221	371	5.465	**<.0001**
Cultural-Provisioning	-0.238	0.252	371	-0.942	0.614
**Regions pairs**					
CESasia-E_Africa	-0.0435	0.386	184	-0.113	1.000
CESasia-Europe	-0.9578	0.300	184	-3.189	**0.043**
CESasia-N_Africa	0.5868	0.767	184	0.766	0.998
CESasia-N_America	-0.2648	0.412	184	-0.643	0.999
CESasia-S_America	0.6222	0.387	184	1.610	0.798
CESasia-S_Africa	0.1255	0.345	184	0.364	1.000
CESasia-WC_Africa	1.0654	0.534	184	1.994	0.550
CESasia-W_Asia	-0.7346	0.350	184	-2.098	0.478
E_Africa-Europe	-0.9143	0.341	184	-2.682	0.162
E_Africa-N_Africa	0.6304	0.792	184	0.796	0.997
E_Africa-N_America	-0.2212	0.432	184	-0.512	1.000
E_Africa-S_America	0.6658	0.430	184	1.549	0.830
E_Africa-S_Africa	0.1691	0.386	184	0.438	1.000
E_Africa-WC_Africa	1.1090	0.556	184	1.995	0.549
E_Africa-W_Asia	-0.6911	0.383	184	-1.806	0.678
Europe-N_Africa	1.5447	0.753	184	2.050	0.511
Europe-N_America	0.6931	0.368	184	1.885	0.625
Europe-S_America	1.5801	0.356	184	4.440	**0.001**
Europe-S_Africa	1.0834	0.296	184	3.662	**0.010**
Europe-WC_Africa	2.0233	0.498	184	4.061	**0.002**
Europe-W_Asia	0.2232	0.304	184	0.735	0.998
N_Africa-N_America	-0.8516	0.813	184	-1.048	0.981

N_Africa-S_America	0.0354	0.791	184	0.045	1.000
N_Africa-S_Africa	-0.4613	0.781	184	-0.591	1.000
N_Africa-WC_Africa	0.4786	0.872	184	0.549	1.000
N_Africa-W_Asia	-1.3215	0.772	184	-1.712	0.739
N_America-S_America	0.8870	0.454	184	1.953	0.578
N_America-S_Africa	0.3903	0.409	184	0.954	0.989
N_America-WC_Africa	1.3302	0.576	184	2.310	0.342
N_America-W_Asia	-0.4699	0.406	184	-1.157	0.964
S_America-S_Africa	-0.4967	0.396	184	-1.255	0.943
S_America-WC_Africa	0.4432	0.567	184	0.782	0.997
S_America-W_Asia	-1.3569	0.405	184	-3.353	**0.027**
S_Africa-WC_Africa	0.9399	0.525	184	1.791	0.688
S_Africa-W_Asia	-0.8602	0.349	184	-2.462	0.258
WC_Africa-W_Asia	-1.8001	0.532	184	-3.382	**0.024**
