# Supplementary material for: The global contribution of vultures towards ecosystem services and sustainability: An experts’ perspective
Source: iScience. 2024 May 7;27(6):109925. doi: 10.1016/j.isci.2024.109925 (PMC11112611; doi:10.1016/j.isci.2024.109925)
Supplement: Document S2. SM_Appendix_S1_Online_Questionnaire [file mmc2.pdf]

Appendix S1. Online Questionnaire form that was circulated to potential vulture experts to be filled, related to all figures and tables in the main text.

Evaluating the ecosystem services associated with vultures and their contribution to the Sustainable Development Goals

Hello, I am Andrea Santangeli (email address: andrea.santangeli [at] helsinki.fi), a post-doc researcher in conservation science at the Finnish Museum of Natural History, University of Helsinki.

All human beings are dependent on ecosystems for their economic and social well-being. Such contributions that people derive from ecosystems are called ecosystem services. They are usually classified as provisioning services (e.g. food and drinking water), regulating services (e.g. carbon sequestration, water purification) and cultural services (e.g. recreation and aesthetic experiences). Vultures (here this term also includes condors) are often associated with one or more ecosystem services, but so far no studies have quantified the potential association of vultures with ecosystem services in a systematic and structured manner. Nor is it clear what scientific evidence underpins such associations.

Moreover, sustainability has become a core focus of international policy agendas, formalized by the Sustainable Development Goals (SDG) of the United Nations. Targets for biodiversity conservation are often associated to sustainability targets. In order to quantify the policy relevance of vulture conservation, it is important to quantify how vulture conservation may help achieve sustainable development.

I thus invite all stakeholders across the globe to fill this questionnaire that will allow evaluating how vultures (including condors) relate to ecosystem services and the Sustainable Development Goals.

I am undertaking this study with the aim to publish the research in an international scientific journal and to disseminate the results among the community of stakeholders involved with vulture and condor work worldwide.
* Required

Participation is voluntary, you are free to leave at any time and your responses are only saved if you submit the form at the end of the questionnaire, and your responses will be kept strictly confidential. The outcomes of this study will be published in a scientific journal and will be shared among this community. Do you consent to take part? *
______

1. Age: ____
2. Sex:
   Female
   Male
   Prefer not to say
   Other:
3. Organisation _____________
4. Highest level of education _____________
5. Occupation _______________ *
6. Region of relevance of your work (please select the region to which your answers are mostly relevant to):*

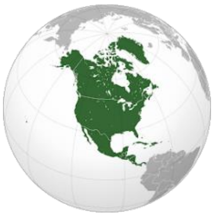

North America

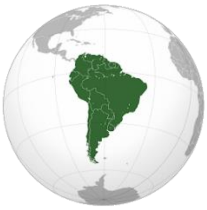

South America

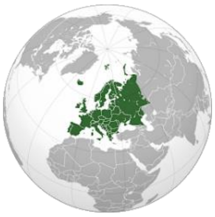

Europe (including the western part of Russia)

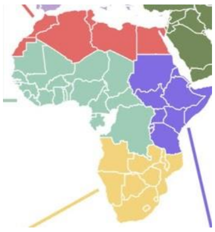

North Africa (red)

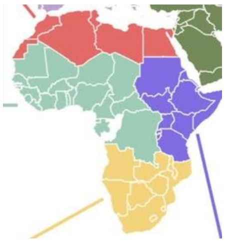

West & Central Africa (light green)

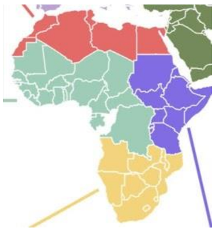

Southern Africa (yellow)

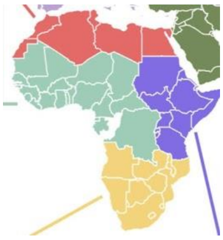

East Africa (purple)

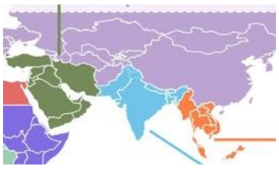
West Asia (green)

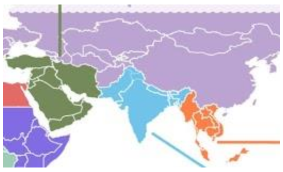
Central & East Asia (light purple)

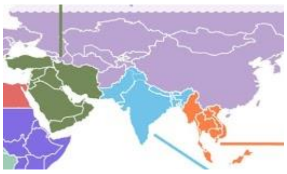
South-East Asia (orange)

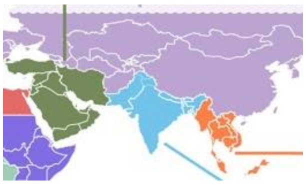
South Asia (light blue)

7. Number of years you have been involved in vulture work: *

8. List the vulture and/or condor species you have been primarily working with during your vulture work years (you can list one or multiple species):

9. Your primary field of expertise: *

Conservation
Ecology
Biology
Social sciences
Veterinary
Forensic sciences
Geography
Agriculture
Economics
Education
Other: ___________________

ECOSYSTEM SERVICES
In this section, you will find a list of ecosystem services covering the three main service groups: provisioning, regulation/maintenance, and cultural services. For each service, please provide a rank following the Likert scale: none, low, moderate, high, very high, based on how relevant vultures are towards each ecosystem service. Your answers should refer to the region you selected above (question 6; i.e. if you listed West Africa, your answers should pertain to West Africa only).

If required, you will find more details on the ecosystem services here: https://cices.eu/

REMEMBER, THIS IS ABOUT ECOSYSTEM SERVICES ASSOCIATED TO VULTURES, NOT OTHER ASPECTS, LIKE THREATS.

10. PROVISIONING - Vulture, or their body parts, used for NUTRITIONAL purposes
None
Low
Moderate
High
Very high

11. PROVISIONING - Vulture, or their body parts, used for NON-nutritional purposes
None
Low
Moderate
High
Very high

12. PROVISIONING - Vultures used for replenishing stock, for breeding, or for genetic stock that can have a value for humans
None
Low
Moderate
High
Very high

13. REGULATION & MAINTENANCE - Vultures aid in decomposing/recycling waste
None
Low
Moderate
High
Very high

14. REGULATION & MAINTENANCE - Vultures aid in reducing smell
None
Low
Moderate
High
Very high

15. REGULATION & MAINTENANCE - Vultures aid in controlling pests and invasive species
None
Low
Moderate
High
Very high

16. REGULATION & MAINTENANCE - Vultures aid in controlling disease
None
Low
Moderate
High
Very high

17. REGULATION & MAINTENANCE - Vultures aid in regulating water conditions
None

Low
Moderate
High
Very high

18. REGULATION & MAINTENANCE - Vultures aid in regulating soil quality
None
Low
Moderate
High
Very high

19. REGULATION & MAINTENANCE - Vultures aid in regulating the global climate
None
Low
Moderate
High
Very high

20. CULTURAL - Vultures enable activities promoting health (e.g. reduce stress) and enjoyment
None
Low
Moderate
High
Very high

21. CULTURAL - Vultures enable the advance of research, knowledge and education on the natural world
None
Low
Moderate
High
Very high

22. CULTURAL - Vultures help people identify with the history or culture of where they live or come from
None
Low
Moderate
High
Very high

23. CULTURAL - Vultures have aesthetic value
None
Low
Moderate
High
Very high

24. CULTURAL - Vultures have symbolic meaning
None
Low
Moderate
High
Very high

25. CULTURAL - Vultures have spiritual importance for people

None
Low
Moderate
High
Very high

26. CULTURAL - Vultures are used to make films or to write books
None
Low
Moderate
High
Very high

27. CULTURAL - Vultures have an existence value, we think they should be conserved
None
Low
Moderate
High
Very high

28. CULTURAL - Vultures should be conserved for the future generations to enjoy or use
None
Low
Moderate
High
Very high

29. Now I ask you to rank the three groups of ecosystem services (Provisioning, Regulation & maintenance, cultural) from the most relevant, medium relevant, and least relevant to vultures relative to your region of expertise as selected in question 8. *

	Most relevant	medium relevant	Least relevant
Provisioning	☐	☐	☐
Regulation & maintenance	☐	☐	☐
Cultural	☐	☐	☐

30. Overall, how would you rank the current scientific evidence underpinning the link between vultures and each ecosystem service group?

	No evidence	weak	moderate	strong	very strong	Don´t know
Provisioning	☐	☐	☐	☐	☐	☐
Regulation & ...	☐	☐	☐	☐	☐	☐
Cultural	☐	☐	☐	☐	☐	☐

31. OPTIONAL QUESTION: Please describe what research you believe should be done in order to better quantify the link between vultures and ecosystem services:

SUSTAINABLE DEVELOPMENT GOALS (SDGs)
In the following section, you will find a list of Sustainable Development Goals (SDGs). For each of them, please provide a rank following the Likert scale: none, low, moderate, high, very high, based on how relevant vultures are towards achieving each Goal. Your answers should refer to the region you selected above (question 6; i.e. if you listed West Africa, your answers should pertain to West Africa only).

If required, you will find more details on the SDGs here:
https://www.undp.org/content/undp/en/home/sustainable-development-goals.html

32. NO POVERTY - Eradicating poverty in all its forms. This involves targeting the most vulnerable, increasing basic resources and services, and supporting communities affected by conflict and climate-related disasters.
None
Low
Moderate
High
Very high

33. ZERO HUNGER - End all forms of hunger and malnutrition, making sure all people–especially children–have sufficient and nutritious food all year. This involves promoting sustainable agricultural, supporting small-scale farmers and equal access to land, technology and markets.
None
Low
Moderate
High
Very high

34. GOOD HEALTH AND WELL-BEING - Good health is essential to sustainable development and the 2030 Agenda reflects the complexity and interconnectedness of the two. It takes into account widening economic and social inequalities, rapid urbanization, threats to the climate and the environment, the continuing burden of HIV and other infectious diseases, and emerging challenges such as noncommunicable diseases. Universal health coverage will be integral to achieving SDG 3, ending poverty and reducing inequalities.
None
Low
Moderate
High
Very high

35. CLEAN WATER AND SANITATION - Safe and affordable drinking water for all by 2030 requires we invest in adequate infrastructure, provide sanitation facilities, and encourage hygiene. Protecting and restoring water-related ecosystems is essential.
None
Low
Moderate
High
Very high

36. SUSTAINABLE CITIES AND COMMUNITIES - Making cities sustainable means creating career and business opportunities, safe and affordable housing, and building resilient societies and economies. It involves investment in public transport, creating green public spaces, and improving urban planning and management in participatory and inclusive ways.
None

Low
Moderate
High
Very high

37. CLIMATE ACTION - Address the needs of developing countries to both adapt to climate change and invest in low-carbon development.
None
Low
Moderate
High
Very high

38. LIFE ON LAND - Human life depends on the earth as much as the ocean for our sustenance and livelihoods. Urgent action must be taken to reduce the loss of natural habitats and biodiversity which are part of our common heritage and support global food and water security, climate change mitigation and adaptation, and peace and security.
None
Low
Moderate
High
Very high

OPTIONAL QUESTION: Please describe what research you believe should be done in order to better quantify the link between vultures and sustainable development:

Thank you for your answers! Your contribution is vital to help better understand the association of vultures with ecosystem services and Sustainable Development!

If you would like to receive a summary report of this study privately by email, please write your email address below (this is totally voluntary): __________________
